# Molecular recognition of planar and non-planar aromatic hydrocarbons through multipoint Ag–π bonding in a dinuclear metallo-macrocycle †
†Electronic supplementary information (ESI) available: Experimental details and characterization data. CCDC 1911739. For ESI and crystallographic data in CIF or other electronic format see DOI: 10.1039/c9sc02619c


**DOI:** 10.1039/c9sc02619c

**Published:** 2019-06-27

**Authors:** Kenichiro Omoto, Shohei Tashiro, Mitsuhiko Shionoya

**Affiliations:** a Department of Chemistry , Graduate School of Science , The University of Tokyo , 7-3-1 Hongo , Bunkyo-ku , Tokyo 113-0033 , Japan . Email: shionoya@chem.s.u-tokyo.ac.jp

## Abstract

Exploration of a novel structural motif of host–guest interactions is one of the most fundamental topics to develop macrocycle-based host–guest/supramolecular systems. Herein, we present an unprecedented mode of inclusion of aromatic hydrocarbons into a macrocyclic cavity *via* multipoint Ag–π bonding as a driving force. A dinuclear Ag^I^-macrocycle encapsulated one molecule of anthracene, a typical planar aromatic hydrocarbon, in solution and in the solid state. Single-crystal X-ray diffraction analysis of the host–guest inclusion complex revealed the binding of anthracene *via* multipoint Ag–π bonding to both Ag^I^ ions arranged within the open-ended nano-cavity of the dinuclear Ag^I^-macrocycle. Notably, this binding motif based on Ag–π bonding was also applied to the inclusion of triptycene, a non-planar aromatic hydrocarbon with a steric tripodal structure, to evaluate the rotational motion of the molecular paddle-wheel in the Ag^I^-macrocycle.

## Abstract 2

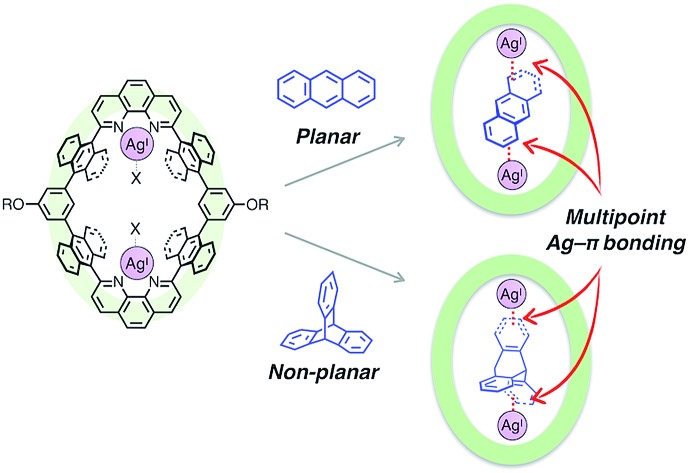
An open-ended cavity of a dinuclear Ag^I^-macrocycle realised an unprecedented recognition mode for planar and non-planar aromatic hydrocarbons *via* multipoint Ag–π bonding.

## Introduction

Since the discovery of crown ether by Pedersen, a great number of excellent examples of functional macrocycles have been reported which can bind guest molecules or ions within their inner spaces.^1^ Guest binding abilities of macrocycles play vital roles in providing various implications to advanced functions in host–guest/supramolecular systems, such as molecular recognition,^2^ activation,^3^ transportation^4^ and fabrication of specific supramolecular architectures (*i.e.* rotaxanes and catenanes).^5^ In general, a variety of non-covalent interactions including hydrogen bonding, coulombic interactions, van der Waals forces and solvophobic effects have been utilised as driving forces for guest uptake of macrocycles.^6^ Besides, metal coordination capable of forming stable, reversible and directional bonds is another binding mode between host and guest compounds, and therefore macrocycles with coordinatively labile metal centres in the cavity have received a lot of attention in recent years in the field of supramolecular chemistry.^7^

**Fig. 1:**
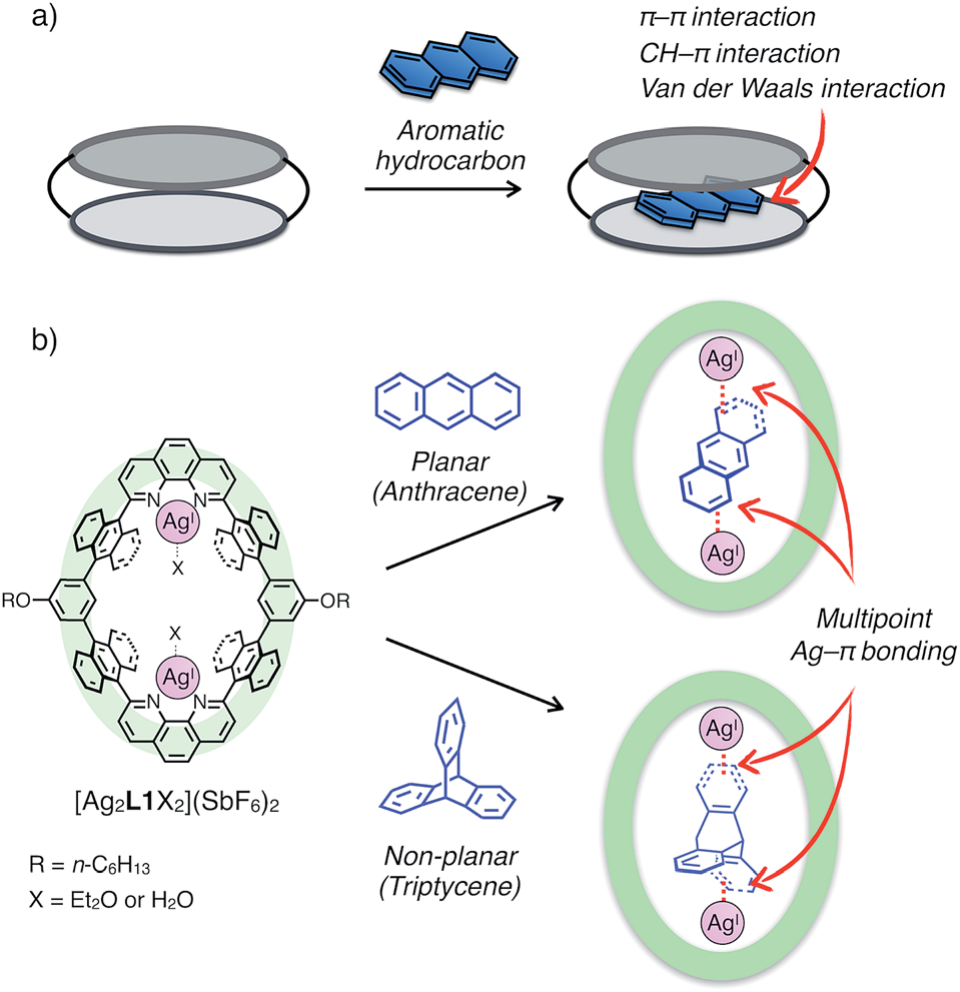
(a) Schematic representation of the binding mode of a non-substituted aromatic hydrocarbon within a macrocyclic host *via* vast-area contacts. (b) The chemical structure of a dinuclear Ag^I^-macrocycle [Ag_2_**L1**X_2_](SbF_6_)_2_ and the schematic representation of the binding modes of planar and non-planar aromatic hydrocarbons *via* multipoint Ag–π bonding.

Aromatic hydrocarbons have been widely investigated as one of the most important classes of guest molecules for macrocycles owing to their ubiquitous structure and electronic properties related to π-conjugation.^8^ In the past few decades, a variety of macrocyclic receptors for aromatic molecules have been reported which can realise recognition,^8^ separation8*c* and regulation8*d* of their properties *via* host–guest interactions. However, in spite of the diversity of macrocyclic structures, molecular recognition of non-substituted, structurally simple aromatic hydrocarbons is still challenging because such hydrocarbons without polar functionalities are difficult to uptake by forming distinct chemical bonds (*i.e.* hydrogen bonds and coordination bonds) with a receptor, and alternatively vast-area contacts through a few weak interactions such as π–π, CH–π and van der Waals interactions are required to encapsulate a pristine aromatic hydrocarbon in a host framework (Fig. 1a). Such vast-area contacts generally need shape complementarity in the host–guest inclusion structure. Therefore, there are limitations in the diversity of host–guest structures, which make it difficult to rationally design a platform for the molecular recognition of non-planar aromatic hydrocarbons with a three-dimensional structure.

Herein, we present an unprecedented mode of inclusion of non-substituted aromatic hydrocarbons into a metallo-macrocycle utilising M–π bonding as a driving force. M–π bonding is a kind of non-Werner type coordination bonding mainly originating from the interactions among d orbitals of metals and π orbitals of aromatic hydrocarbons.^9^–^11^ In general, M–π bonding has higher directionality and site-specificity than the above-mentioned conventional intermolecular interactions, and the resulting M–π complexes have the potential to exhibit unique functions based on the chemical and physical properties of the metal centres. Therefore, metallo-macrocycles possessing a nano-space with multiple metal centres for M–π bonding are expected to provide a novel structural motif to recognise aromatic guest molecules, in which the inclusion structure and the guest selectivity can be affected by the type, number, and arrangement of the inner metal ions.^12^ Based on this concept, we have recently developed a dinuclear Ag^I^-macrocycle [Ag_2_**L1**X_2_](SbF_6_)_2_ (**L1** = macrocyclic ligand and X = Et_2_O or H_2_O) which possesses an anthracene-based rhombic structure with a nano-space with two Ag^I^ centres (Fig. 1b). In this system, the Ag^I^-macrocycle can accommodate a ditopic aromatic molecule such as cyclophanes and ferrocene utilising multiple Ag–π bonds as a main driving force.12a,c Herein we report that the Ag^I^-macrocycle effectively recognised an anthracene molecule, a planar aromatic hydrocarbon, in the open-ended cavity through multiple Ag–π bonds with both ends of anthracene (Fig. 1b). Moreover, we found that this guest-binding motif based on Ag–π bonding does not require vast-area contacts between the host and guest, which achieved the binding of a non-planar aromatic hydrocarbon, triptycene, in a similar manner.

## Results and discussion

**Fig. 2:**
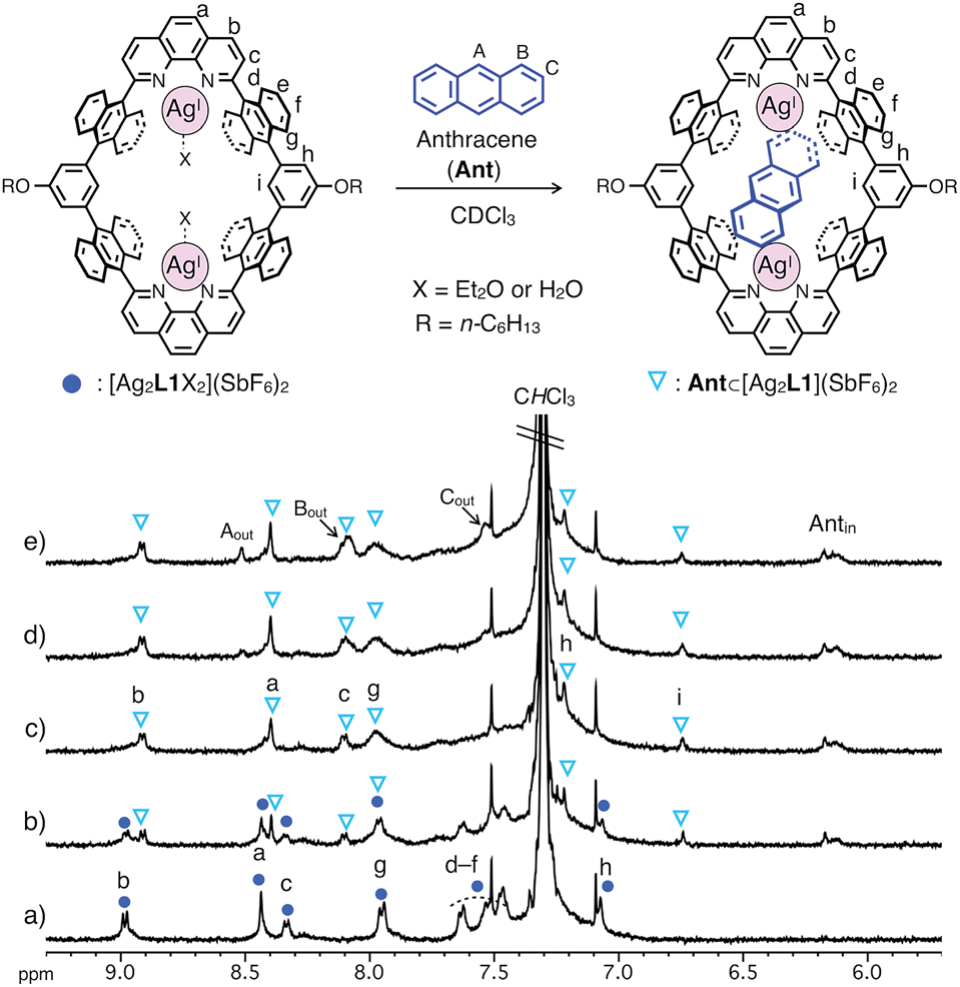
Partial ^1^H NMR spectra of [Ag_2_**L1**X_2_](SbF_6_)_2_ (0.07 mM) in the presence of (a) 0.0, (b) 0.5, (c) 1.0, (d) 1.5 and (e) 2.0 eq. of anthracene (**Ant**) (500 MHz, CDCl_3_, 220 K). Ant_in_ represents the signals of the included **Ant**.

The binding of anthracene (**Ant**) to a dinuclear Ag^I^-macrocycle [Ag_2_**L1**X_2_](SbF_6_)_2_ was first investigated by ^1^H NMR titration experiments at 220 or 300 K and mass spectrometry. Upon the addition of **Ant** to a solution of [Ag_2_**L1**X_2_](SbF_6_)_2_ (0.07 mM) in CDCl_3_, the ^1^H NMR signals of [Ag_2_**L1**X_2_](SbF_6_)_2_ at 220 K were gradually replaced by a new set of signals with an increased amount of **Ant** (Fig. 2a–c). In the presence of more than 1.0 eq. of **Ant**, the original signals were almost completely replaced by a new set of signals, and the signals of free **Ant** (A_out_, B_out_ and C_out_) appeared at around 7.5–8.6 ppm, suggesting the formation of a 1 : 1 host–guest inclusion complex, **Ant**⊂[Ag_2_**L1**](SbF_6_)_2_ (Fig. 2c–e). The new signals observed around the higher magnetic field region (Ant_in_ around 6.0–6.2 ppm) can be assigned to the included **Ant**, which are significantly up field shifted (|Δ*δ*| = *ca.* 1.4–2.4 ppm) from their original positions due to the shielding effect from the anthracene walls of [Ag_2_**L1**]^2+^.^13^

Notably, at a temperature as high as 300 K, as the intermolecular exchange between bound and free **Ant** molecules is faster than the timescale of ^1^H NMR spectroscopy, ^1^H NMR titration experiments at 300 K resulted in the sequential shift of the signals of [Ag_2_**L1**X_2_](SbF_6_)_2_ due to host–guest interactions (Fig. S1 and S2†). From the curve-fitting of the ^1^H NMR data at 300 K based on the formation of a 1 : 1 host–guest complex, the binding constant between **Ant** and the Ag^I^-macrocycle (*K*_a_(**Ant**) = [**Ant**⊂[Ag_2_**L1**]^2+^]/([[Ag_2_**L1**X_2_]^2+^][**Ant**]) M^–1^) was determined to be (3.0 ± 0.4) × 10^4^ M^–1^ in CDCl_3_ at 300 K (Fig. S3†), which is comparable to those of already reported examples of macrocyclic receptors for aromatic hydrocarbons.8c,f,^14^ The formation of a 1 : 1 host–guest complex was also supported by ESI-TOF mass measurements (*m*/*z* = 903.72 as **Ant**⊂[Ag_2_**L1**]^2+^, Fig. S6†).

**Fig. 3:**
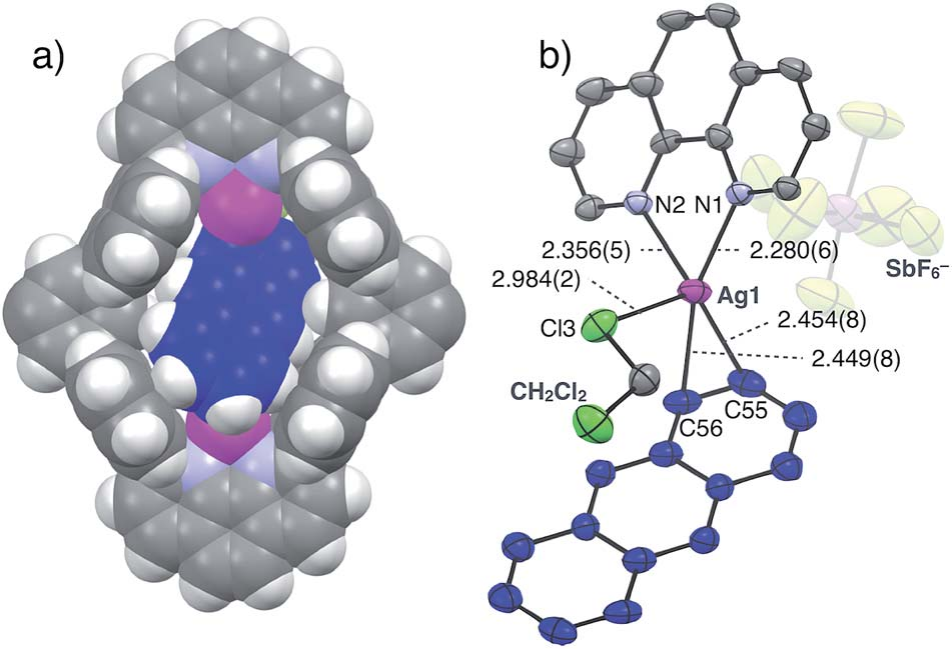
Crystal structure of **Ant**⊂[Ag_2_**L1**(CH_2_Cl_2_)_2_](SbF_6_)_2_. (a) Space filling model and (b) ORTEP view (50% probability level) of the partial structure (solvents, side-alkyloxy chains and counter anions in (a) are omitted for clarity). Ag: magenta, C: grey, C of **Ant**: blue, Cl: pale green, F: yellow, H: white, N: light blue and Sb: purple.

The structure and binding mode of the resulting complex were finally determined by single-crystal X-ray analysis (Fig. 3). By slow *n*-pentane vapour diffusion into a mixture of **L1**, AgSbF_6_ (4.2 eq.) and **Ant** (10 eq.) in CH_2_Cl_2_ in the dark at room temperature, yellow block crystals suitable for single-crystal X-ray analysis were obtained. In the resulting crystal structure, one molecule of **Ant** was included in the cavity of [Ag_2_**L1**]^2+^*via* η^2^-type Ag–π bonding at both terminal edges of the elongated π-surface in an *anti*-manner (Fig. 3a). These Ag^I^ ions were in a distorted square pyramidal five-coordinate geometry with two N-atoms of phenanthroline, two C-atoms of the included **Ant**, and one Cl-atom of a coordinating solvent: CH_2_Cl_2_ (Ag–N1 2.280(6) Å; Ag–N2 2.356(5) Å; Ag–C55 2.454(8) Å; Ag–C56 2.449(8) Å; Ag–Cl3 2.984(2) Å, Fig. 3b). These observations suggest that multipoint Ag–π bonding between **Ant** and Ag^I^ ions, which are precisely arranged on the inner surface of the nano-space, works as an effective driving force to bind **Ant**. Multipoint CH–π interactions between the H-atoms of the included **Ant** and the π-surface of anthracene-walls of the macrocycle may also contribute to stabilise the resulting complex as well. Notably, **Ant**⊂[Ag_2_**L1**(CH_2_Cl_2_)_2_]^2+^ possesses a *C*_i_-symmetrical structure in the crystal due to the inclination of the included **Ant**. On the other hand, the ^1^H NMR spectrum of **Ant**⊂[Ag_2_**L1**](SbF_6_)_2_ showed a simple set of ^1^H NMR signals (Fig. 2b–e) suggesting a more symmetrical (*D*_2h_) structure with successive fluxional movement of **Ant** in the nano-cavity *via* intramolecular Ag–π exchange.

Based on these results, the binding properties of [Ag_2_**L1**X_2_](SbF_6_)_2_ to other aromatic hydrocarbons of different sizes were examined. We previously reported that a smaller aromatic hydrocarbon, *p*-xylene, formed not a 1 : 1 but a 2 : 1 complex, (*p*-xylene)_2_⊂[Ag_2_**L1**]^2+^, in the crystalline state.12*a* On the other hand, a ^1^H NMR titration experiment using *p*-xylene showed negligible shift in the host signals (|Δ*δ*| < 0.04 ppm) even after adding 4 eq. of *p*-xylene, suggesting a lower affinity to [Ag_2_**L1**X_2_](SbF_6_)_2_ or a different binding mode from that of **Ant**⊂[Ag_2_**L1**](SbF_6_)_2_ in solution (Fig. S22 and S24†). Similarly, naphthalene, which has an intermediate size between *p*-xylene and **Ant**, showed a smaller shift in the host signals (|Δ*δ*| < 0.02 ppm) even after adding 4 eq. of naphthalene in the ^1^H NMR titration experiment at 300 K (Fig. S23† and S24†). These results may reflect the insufficiency of the molecular size of *p*-xylene and naphthalene to simultaneously form Ag–π bonds at both Ag^I^ centres arranged in the macrocyclic skeleton. These results suggest the importance of the arrangement mode of Ag^I^ ions in the macrocycle in controlling the stability, selectivity and structure of the resulting host–guest complex.

**Fig. 4:**
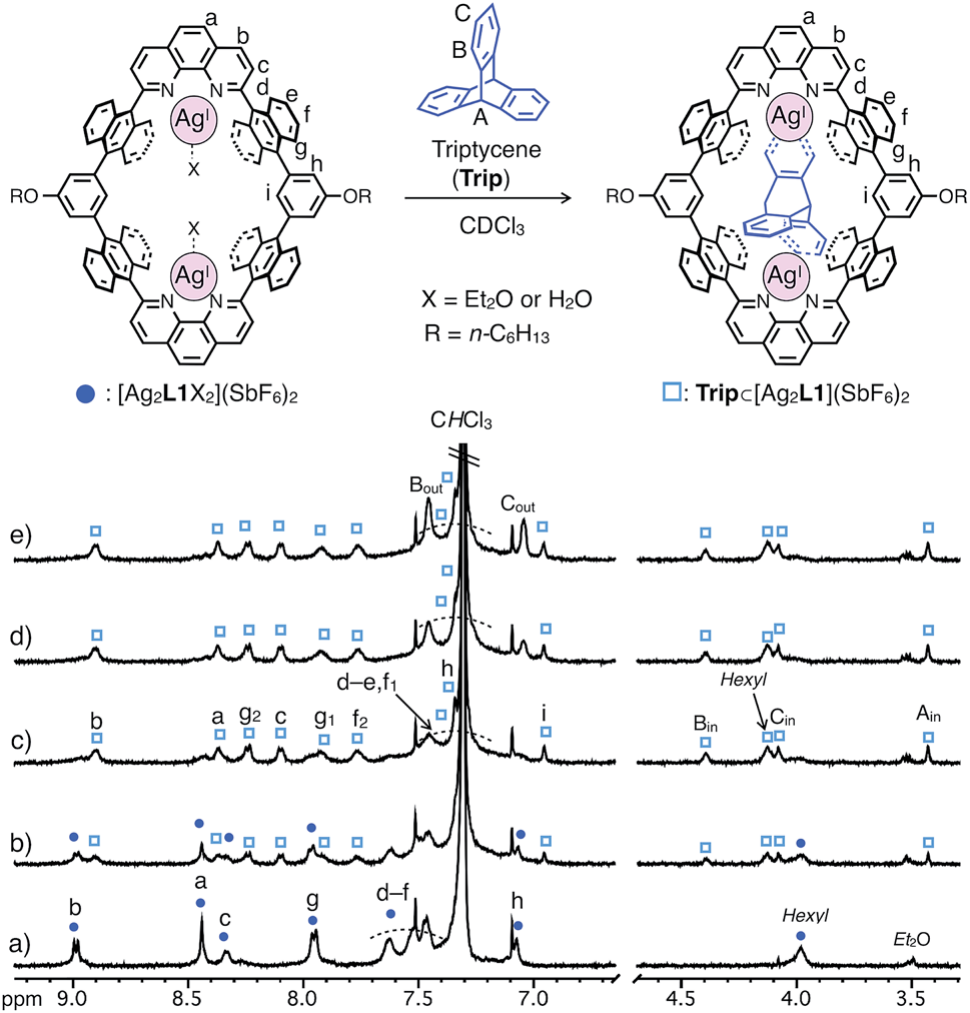
Partial ^1^H NMR spectra (500 MHz, CDCl_3_, 220 K) of [Ag_2_**L1**X_2_](SbF_6_)_2_ (0.11 mM) in the presence of (a) 0.0, (b) 0.5, (c) 1.0, (d) 1.5 and (e) 2.0 eq. of triptycene (**Trip**). *Hexyl* represents the signal of side-alkyloxy chains of **L1**.

In contrast, triptycene (**Trip**), which is a non-planar aromatic hydrocarbon with a steric tripodal structure, was found to be effectively included within the cavity of [Ag_2_**L1**X_2_](SbF_6_)_2_. The inclusion behavior of **Trip** was revealed by a ^1^H NMR titration experiment in CDCl_3_ at 220 K, in which the host signals were replaced by a new set of signals in the presence of more than 1.0 eq. of **Trip** to form an 1 : 1 host–guest complex **Trip**⊂[Ag_2_**L1**](SbF_6_)_2_ (Fig. 4).^13^ The signals of the included **Trip** (B_in_) showed a strong rotating frame Overhauser effect (ROE) correlation with the proton inside [Ag_2_**L1**]^2+^ (H_i_), which suggests the existence of **Trip** within the nano-cavity of the macrocycle (Fig. S18†). The formation of **Trip**⊂[Ag_2_**L1**](SbF_6_)_2_ was also supported by ESI-TOF mass analysis (*m*/*z* = 941.21 as **Trip**⊂[Ag_2_**L1**]^2+^, Fig. S20†).

**Fig. 5:**
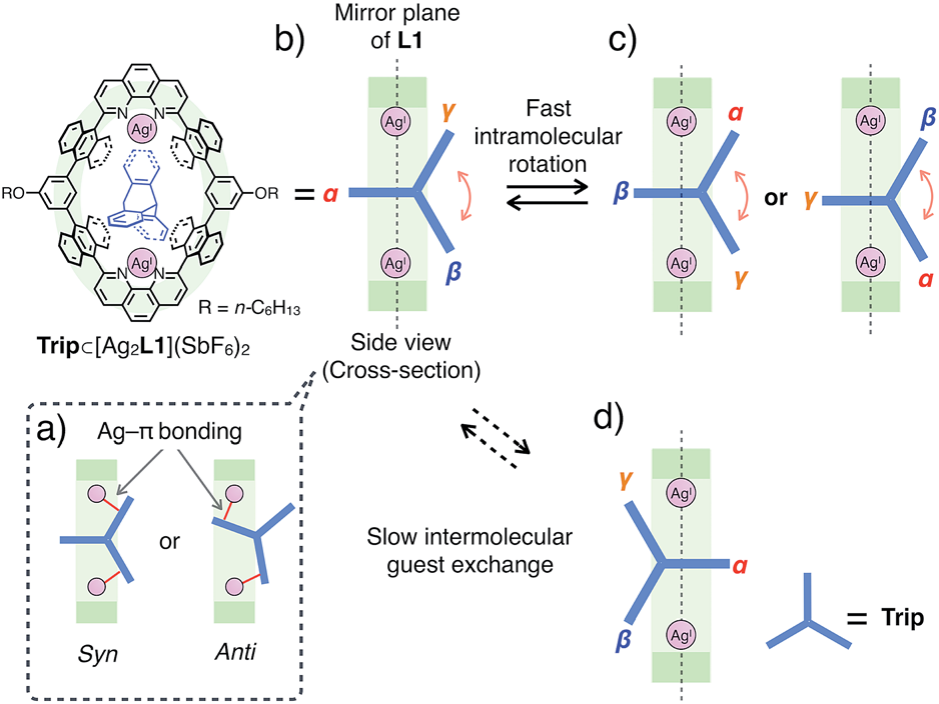
Schematic representation of the plausible binding modes and molecular dynamics of **Trip**⊂[Ag_2_**L1**](SbF_6_)_2_. (a) Plausible side cross-sectional views of **Trip**⊂[Ag_2_**L1**](SbF_6_)_2_ where Ag^I^ ions coordinate to the π-planes of **Trip** in an *anti* or a *syn* manner. (b–d) Plausible side cross-sectional views of the rotational motion of **Trip**, where **Trip** exhibits faster intramolecular rotational motion (b and c) and a slower intermolecular guest exchange (d) than the timescale of ^1^H NMR observation.

Similar to the case of **Ant**, the rates of intermolecular exchange of bound and free **Trip** at a higher temperature (300 K) became faster than the timescale of ^1^H NMR, which resulted in the observation of a dynamically averaged ^1^H NMR spectrum (Fig. S9 and S10†). From the curve-fitting of the ^1^H NMR data of the guest titration experiment at 300 K based on the formation of a 1 : 1 host–guest complex, the binding constant between **Trip** and the Ag^I^-macrocycle (*K*_a_(**Trip**) = [**Trip**⊂[Ag_2_**L1**]^2+^]/([[Ag_2_**L1**X_2_]^2+^][**Trip**]) M^–1^) was determined to be (3.1 ± 0.2) × 10^4^ M^–1^ in CDCl_3_ at 300 K (Fig. S11†), which is comparable to that of **Ant** (*K*_a_(**Ant**) = (3.0 ± 0.4) × 10^4^ M^–1^).^14^ This result suggests host–guest interactions between **Trip** and Ag^I^-macrocycle similar to that of **Ant** and Ag^I^-macrocycle, where multipoint Ag–π bonding simultaneously formed at both ends of the aromatic molecule works as a dominant driving force for guest binding. Molecular modeling of **Trip**⊂[Ag_2_**L1**]^2+^ based on the crystal structure of the aforementioned **Ant**⊂[Ag_2_**L1**](SbF_6_)_2_ (Fig. 3) and that of a previously reported Ag^I^ complex of **Trip** supports the multipoint binding of **Trip**, where Ag^I^ ions coordinate to the π-planes of **Trip** in the *anti* or *syn* form (Fig. 5a and S21†).10d,g,h Such a binding motif of **Trip** suggests that the open-ended nano-cavity of [Ag_2_**L1**X_2_](SbF_6_)_2_ with two Ag^I^ ions would work as an effective receptor for not only planar but also non-planar aromatic molecules which allows multipoint Ag–π bonding.

Notably, the included **Trip** exhibits rotational motion at 220 K, which affects the symmetry of the resulting host–guest complex, **Trip**⊂[Ag_2_**L1**]^2+^ (Fig. 5b–d).^15^ Upon binding of **Trip**, the ^1^H NMR signals of the protons of anthracene walls (H_g_ in Fig. 4a) of the macrocycle split into two separate signals (H_g1_ and H_g2_ in Fig. 4c), suggesting the desymmetrisation of the host skeleton from *D*_2h_-symmetry to *C*_2v_-symmetry (loss of the mirror plane on the cyclic skeleton of **L1**). On the other hand, the three aromatic panels of the included **Trip** (α, β and γ in Fig. 5b and c) were observed as two identical signals in the higher magnetic field region (B_in_ and C_in_ at 4.4 and 4.1 ppm in Fig. 4c), suggesting conservation of the *D*_3h_-symmetry of the pristine tripodal structure of **Trip** even within the nano-cavity of the macrocycle. Considering such a respective loss and conservation of the symmetry of the host and the guest, the included **Trip** was supposed to be deviated from the mirror plane of the cyclic skeleton of the macrocycle **L1** without fast intermolecular guest exchange to desymmetrise the host skeleton (Fig. 5d). Besides, the included **Trip** was supposed to exhibit fast intramolecular rotational movement around the 3-fold axis of the tripodal structure to average the magnetic environment of the three aromatic panels in the timescale of ^1^H NMR observation (α, β and γ in Fig. 5b and c). Such a specific rotational motion of **Trip** bound to two Ag^I^ ions suggests that the open-ended nano-cavity of [Ag_2_**L1**X_2_](SbF_6_)_2_ is suitable to bind aromatic guest molecules and to maintain and regulate the degree of freedom of their molecular motion, which potentially plays a key role as functional molecular machines.

## Conclusions

In this study, host–guest interactions between a dinuclear Ag^I^-macrocycle [Ag_2_**L1**X_2_](SbF_6_)_2_ and aromatic hydrocarbons were investigated. [Ag_2_**L1**X_2_](SbF_6_)_2_ has an ability to strongly include not only a planar (**Ant**) but also a non-planar (**Trip**) aromatic hydrocarbon which allows multipoint Ag–π bonding at both Ag^I^ centres simultaneously. Such a Ag^I^-based specific binding motif is quite distinct from those of conventional macrocyclic receptors for aromatic molecules which require close and vast-area contacts between the aromatic guest and the nano-cavity of the macrocycle. Such a unique binding motif utilising non-Werner type M–π coordination would provide us an important clue to the rational design of the structures, properties and metal-centred functions of the resulting host–guest complexes. This finding would help to develop a new dimension to macrocycle-based supramolecular chemistry and molecular machines based on the selectivity, reactivity and dynamics of M–π bonding within a confined space of metallo-macrocycles.

## Conflicts of interest

There are no conflicts to declare.

## Supplementary Material

Supplementary information

Crystal structure data
