# Supplementary material for: Molecular recognition of planar and non-planar aromatic hydrocarbons through multipoint Ag–π bonding in a dinuclear metallo-macrocycle
Source: Chem Sci. 2019 Jun 27;10(30):7172–6. doi: 10.1039/c9sc02619c (PMC6764282; doi:10.1039/c9sc02619c)
Supplement: Supplementary file 1 [file SC-010-C9SC02619C-s001.pdf]

Electronic Supplementary Material (ESI) for Chemical Science.
This journal is © The Royal Society of Chemistry 2019

Electronic Supplementary Information (ESI)

Molecular recognition of planar and non-planar aromatic hydrocarbons through multipoint Ag–π bonding in a dinuclear metallo-macrocycle

Kenichiro Omoto, Shohei Tashiro and Mitsuhiko Shionoya*

Department of Chemistry, Graduate School of Science, The University of Tokyo
7-3-1 Hongo, Bunkyo-ku, Tokyo 113-0033, Japan

1. Abbreviation page S2
2. Materials and methods page S2
3. Guest binding behaviors of [Ag_2L1X_2](SbF_6)$_2$ page S3
4. References page S17

1. Abbreviation

COSY: correlated spectroscopy, Et_2O: diethyl ether, ESI-TOF: electrospray ionization-time-of-flight, THF: tetrahydrofurane, NMR: nuclear magnetic resonance, TMS: tetramethylsilane, XRD: X-ray diffraction, PXRD: powder X-ray diffraction, DSC: differential scanning calorimetry,

2. Materials and methods

All solvents, organic and inorganic reagents are commercially available, and were used without further purification. Macrocyclic ligand **L1**, dinuclear Ag^{I}-complex $[Ag_2$**L1**$X_2](SbF_6)_2$ (X = Et_2O or H_2O) were prepared according to previously reported procedures.[1]

NMR spectroscopic measurements were performed using a Bruker AVANCE 500 (500 MHz for ^{1}H) spectrometer. NMR spectra were calibrated as below; tetramethylsilane ($Si(CH_3)_4$) = 0 ppm for ^{1}H in $CDCl_3$. *p*-Dimethoxybenzene was added as an internal standard for the calibration of the concentration of samples. ESI-TOF mass spectra were recorded on a Micromass LCT spectrometer and a Micromass LCT Premier spectrometer. Single-crystal X-ray crystallographic analyses were performed using a Rigaku RAXIS-RAPID imaging plate diffractometer with MoKα radiation, and the obtained data were calculated using a CrystalStructure crystallographic software package except for refinement, which was performed using SHELXL-97.[2] Molecular modeling was performed by a Spartan'08 based on MMFF97 as a force field.

3. Guest binding behaviors of $[Ag_2L1X_2](SbF_6)_2$

<u>Complexation of $[Ag_2L1X_2](SbF_6)_2$ and anthracene</u>

^{1}H NMR titration experiment at 300 K

To a solution of $[Ag_2\mathbf{L1}X_2](SbF_6)$ in $CDCl_3$ (0.11 mM, 475 μL, 0.052 μmol, 1.0 eq) was added a solution of anthracene (**Ant**) in $CDCl_3$ (20 mM). Curve fitting of the obtained data determined a stability constant $K_a(\mathbf{Ant}) = [\mathbf{Ant}\subset[Ag_2\mathbf{L1}]^{2+}]/([\mathbf{Ant}][[Ag_2\mathbf{L1}X_2]^{2+}])$ to be $(3.0 \pm 0.4) \times 10^4$ M^{-1} in $CDCl_3$ at 300 K.

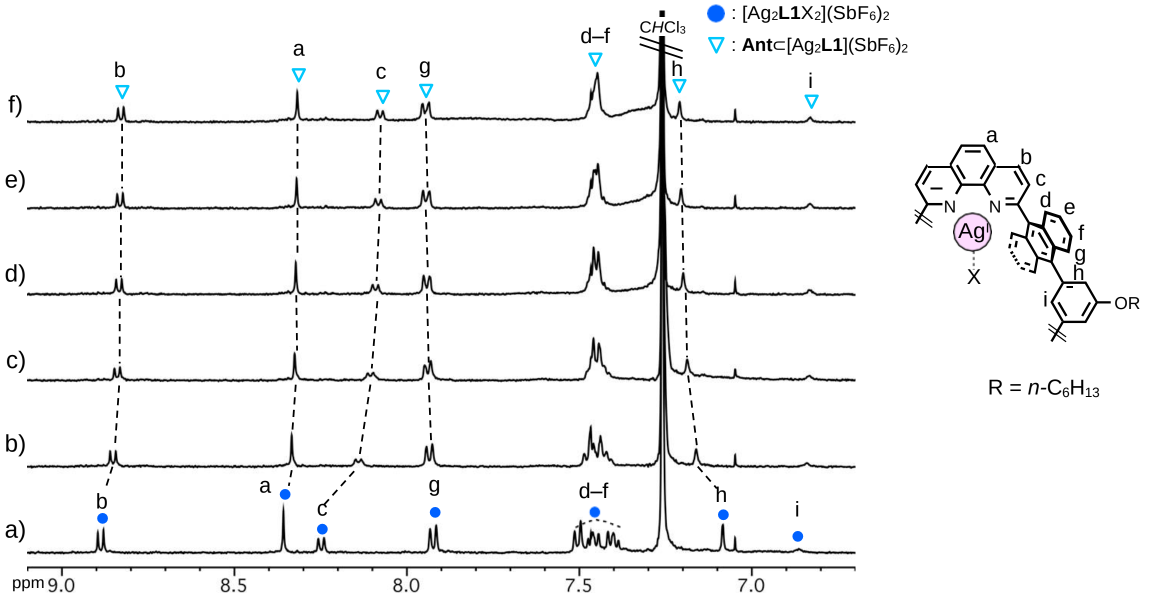

Fig. S1. Partial ^{1}H NMR spectra of $[Ag_2\mathbf{L1}X_2](SbF_6)_2$ (0.11 mM) in the presence of a) 0.0, b) 1.0, c) 2.0, d) 3.0, e) 4.0, and f) 5.0 eq of **Ant** (500 MHz, $CDCl_3$, 300 K).

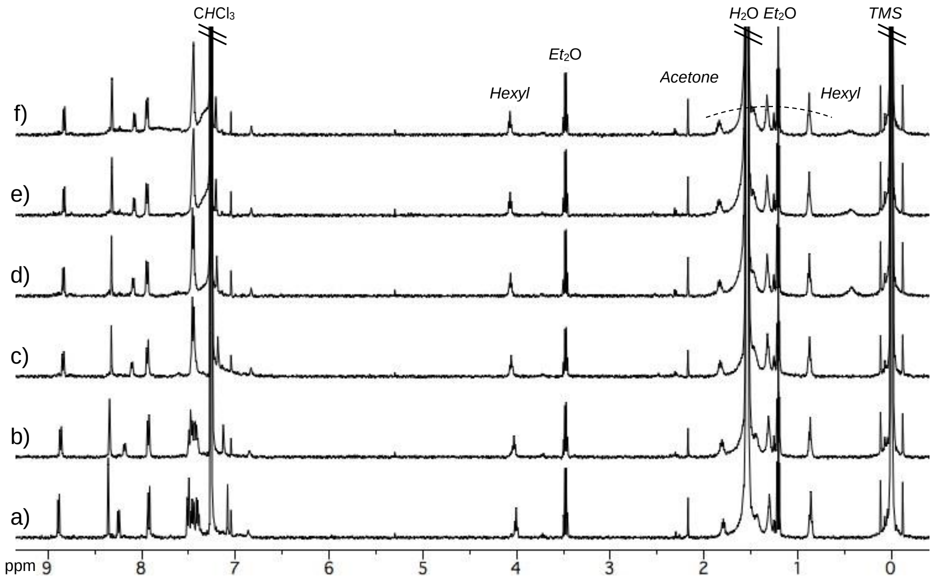

Fig. S2. ^{1}H NMR spectra of $[Ag_2\mathbf{L1}X_2](SbF_6)_2$ (0.11 mM) in the presence of a) 0.0, b) 1.0, c) 2.0, d) 3.0, e) 4.0, and f) 5.0 eq of **Ant** (500 MHz, $CDCl_3$, 300 K).

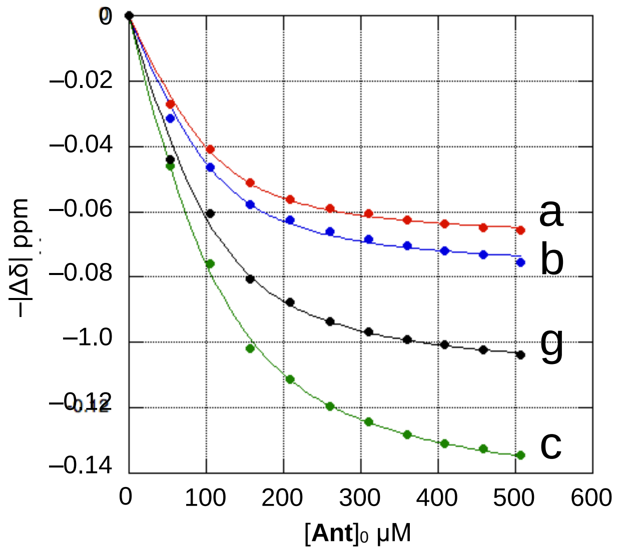

$$K_a(\mathbf{Ant}) = \frac{[\mathbf{Ant}\subset[Ag_2\mathbf{L1}]^{2+}]}{[\mathbf{Ant}]\ [[Ag_2\mathbf{L1}X_2]^{2+}]}$$

$$= (3.0 \pm 0.4) \times 10^{4}\ M^{-1}$$

in $CDCl_3$ at 300 K

Fig. S3. Stability constant analysis by the least square fitting to the shift of NMR signals ($H_{a\text{–}c,\ g}$) in the titration experiment described in Figs. S1–S2 (solid circles: observed, lines: calculated). [**Ant**]$_0$indicates the initial concentration of **Ant**.

^{1}H NMR titration experiment at 220 K

To a solution of [Ag_2**L1**X_2](SbF_6)$_2$ in $CDCl_3$ (0.07 mM, 450 μL, 0.029 μmol, 1.0 eq) was added a solution of anthracene (**Ant**) in $CDCl_3$ (20 mM).

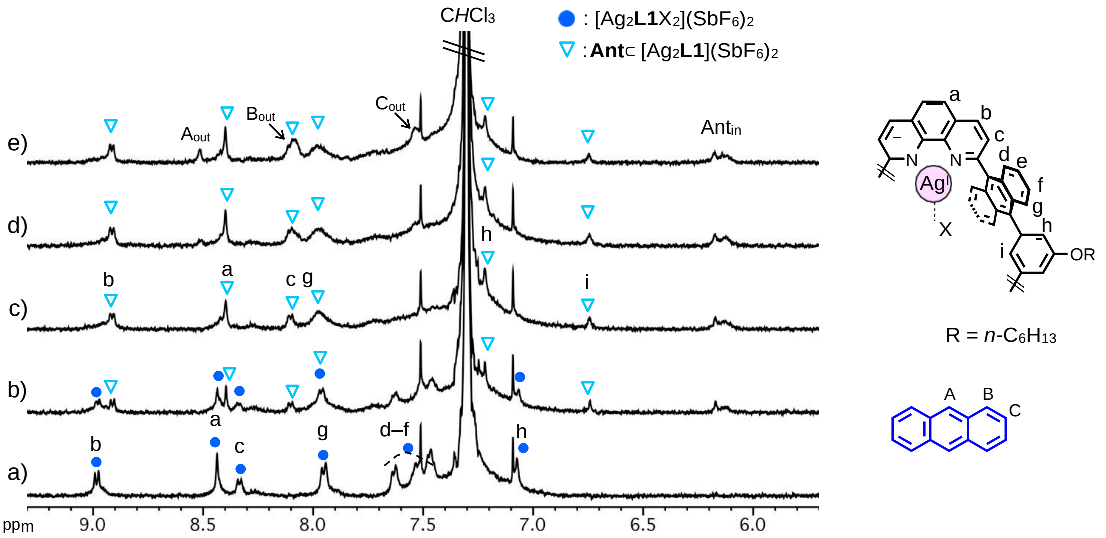

Fig. S4. Partial ^{1}H NMR spectra of [Ag_2**L1**X_2](SbF_6)$_2$ (0.07 mM) in the presence of a) 0.0, b) 0.5, c) 1.0, d) 1.5, and e) 2.0 eq of **Ant** (500 MHz, $CDCl_3$, 220 K). **Ant$_{in}$** represents the signals of included **Ant**.

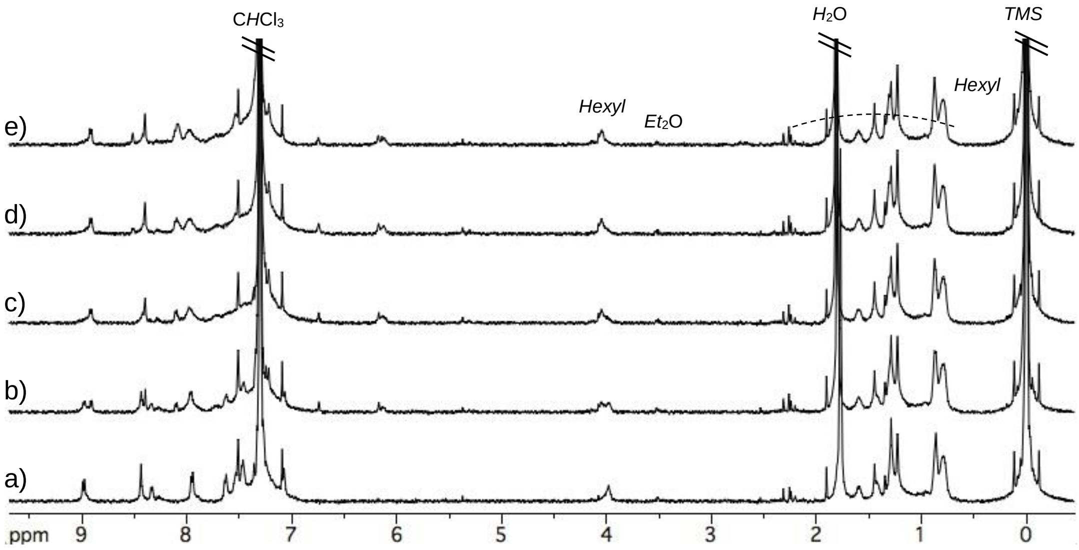

Fig. S5. ^{1}H NMR spectra of [Ag_2**L1**X_2](SbF_6)$_2$ (0.07 mM) in the presence of a) 0.0, b) 0.5, c) 1.0, d) 1.5, and e) 2.0 eq of **Ant** (500 MHz, $CDCl_3$, 220 K).

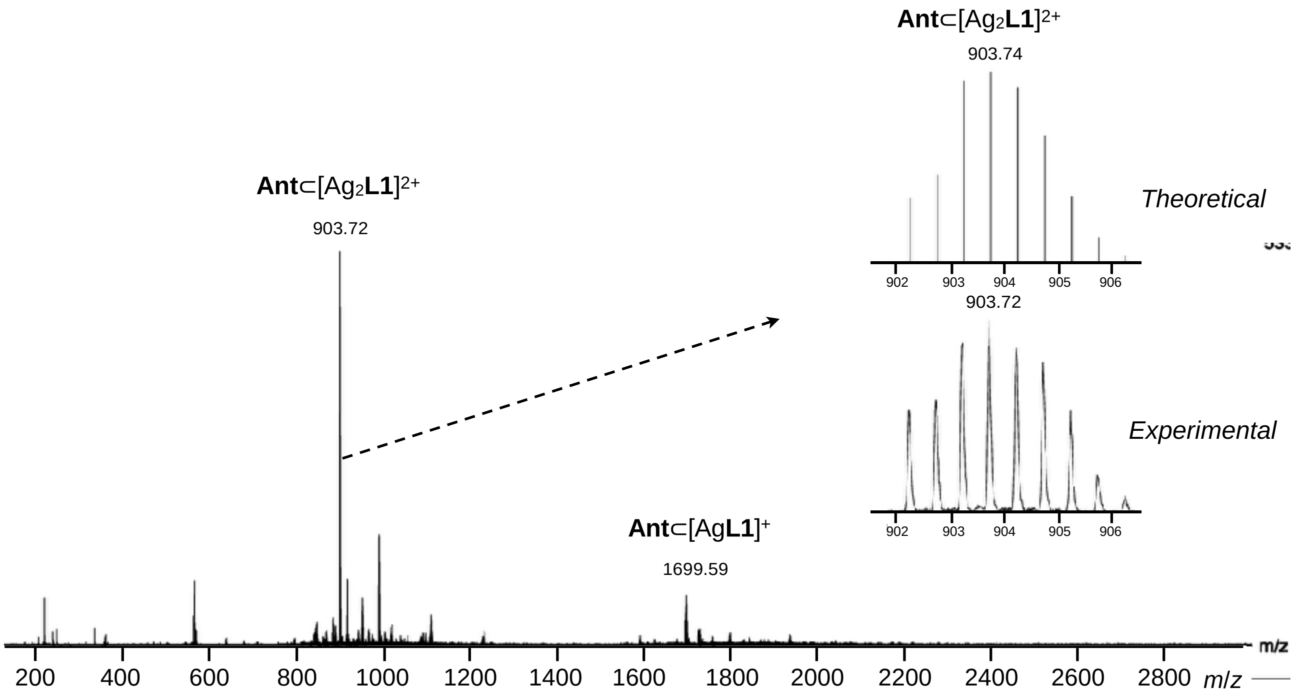

Fig. S6. ESI-TOF mass spectrum of a mixture of $[Ag_2\mathbf{L1}X_2](SbF_6)_2$ and 5.0 eq of **Ant** in $CHCl_3$.

Crystallization of Ant⊂$[Ag_2L1(CH_2Cl_2)_2](SbF_6)_2{\cdot}(C_5H_{12})_2{\cdot}(CH_2Cl_2)_2$

To a suspension of **L1** (0.23 mM, 450 μL, 0.10 μmol, 1.0 eq) in $CHCl_3$ was added a solution of $AgSbF_6$ (200 mM, 2.1 μL, 0.42 μmol, 4.2 eq) in acetone and a solution of **Ant** (200 mM, 5.2 μL, 1.0 μmol, 10 eq) in $CHCl_3$ to obtain a clear yellow solution. The solvent was once removed by evaporation under reduced pressure. Then a resulting solid was dissolved in CH_2Cl_2 (250 μL). Yellow brock crystals suitable for single crystals XRD measurement were obtained after *n*-pentane vapor diffusion in the dark over about 10 days.

Crystal data of Ant⊂$[Ag_2L1(CH_2Cl_2)_2](SbF_6)_2{\cdot}(C_5H_{12})_2{\cdot}(CH_2Cl_2)_2$

Crystal data of $C_{132}H_{118}Ag_2Cl_8F_{12}N_4O_2Sb_2$: F_w = 2763.25, crystal dimensions 0.30 × 0.30 × 0.10 mm^3, monoclinic, space group $P2_1/c$, a = 18.8596(8), b = 13.4589(6), c = 23.972(1) Å, β = 99.096(1)°, V = 6008.3(5) Å^3, Z = 2, ρ_{calcd} = 1.527 g cm^{-3}, μ = 1.0146 mm^{-1}, T = 93 K, λ(MoKα) = 0.71075 Å, $2\theta_{max}$ = 50.0°, 46007/10551 reflection collected/unique (R_{int} = 0.0485), R_1 = 0.0773 ($I > 2\sigma(I)$), wR_2 = 0.2257 (for all data), GOF = 1.025, largest diff. peak and hole 2.84/–1.48 eÅ^{-3}. CCDC deposit number 1911739.

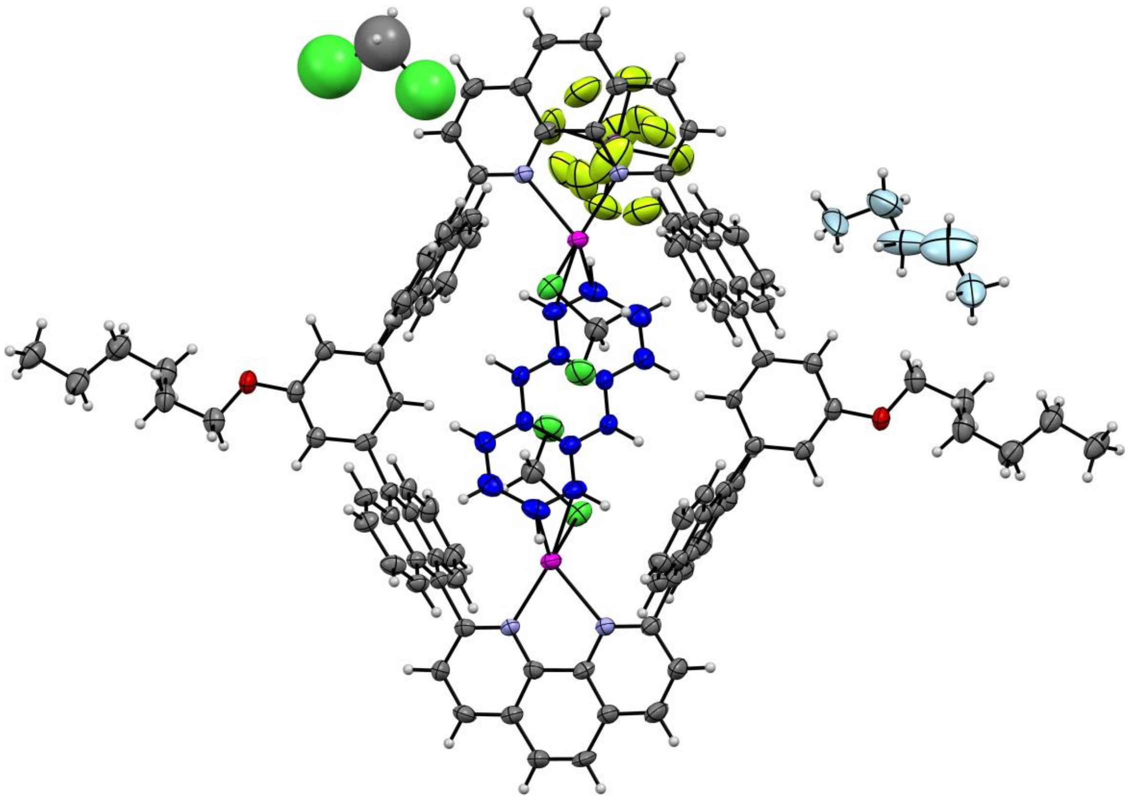

Fig. S7. ORTEP view (50% probability level) of **Ant**⊂[Ag_2**L1**$(CH_2Cl_2)_2$]$(SbF_6)_2$·$(C_5H_{12})_2$·$(CH_2Cl_2)_2$. (Ag: magenta, C: grey, C of **Ant**: blue, C of *n*-pentane: pale blue, Cl: pale green, F: yellow, H: white, N: blue, O: red, Sb: purple)

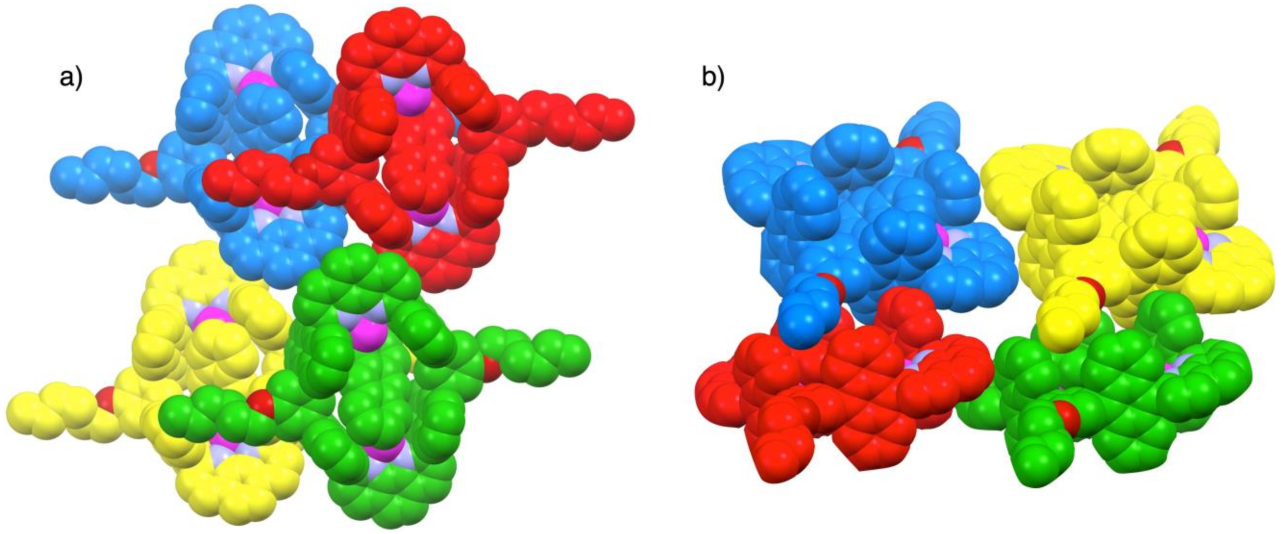

Fig. S8. Crystal packing of **Ant**⊂[Ag_2**L1**$(CH_2Cl_2)_2$]$(SbF_6)_2$·$(C_5H_{12})_2$·$(CH_2Cl_2)_2$. (hydrogen atoms, solvents, and counter anions are omitted for clarity). Views from a) *b* axis and b) *c* axis.

Complexation of $[Ag_2L1X_2](SbF_6)_2$ and triptycene

^{1}H NMR titration experiment at 300 K

To a solution of $[Ag_2\mathbf{L1}X_2](SbF_6)_2$ in $CDCl_3$ (0.07 mM, 400 μL, 0.03 μmol, 1.0 eq) was added a solution of triptycene (**Trip**) in $CDCl_3$ (40 mM). Curve fitting of the obtained data determined a stability constant K_a(**Trip**) = $[\mathbf{Trip}\subset[Ag_2\mathbf{L1}]^{2+}]/([\mathbf{Trip}][[Ag_2\mathbf{L1}X_2]^{2+}])$ to be $(3.1 \pm 0.2) \times 10^4$ M^{-1} in $CDCl_3$ at 300 K.

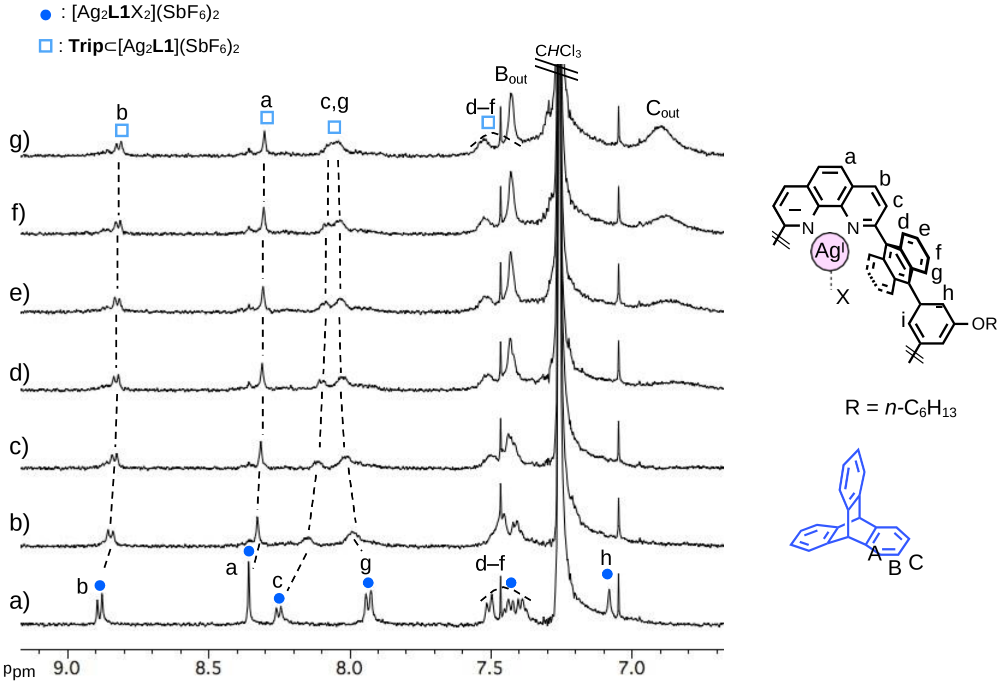

Fig. S9. Partial ^{1}H NMR spectra of $[Ag_2\mathbf{L1}X_2](SbF_6)_2$ (0.07 mM) in the presence of a) 0.0, b) 1.0, c) 2.0, d) 3.0, e) 4.0, f) 5.0, and g) 7.0 eq of **Trip** (500 MHz, $CDCl_3$, 300 K).

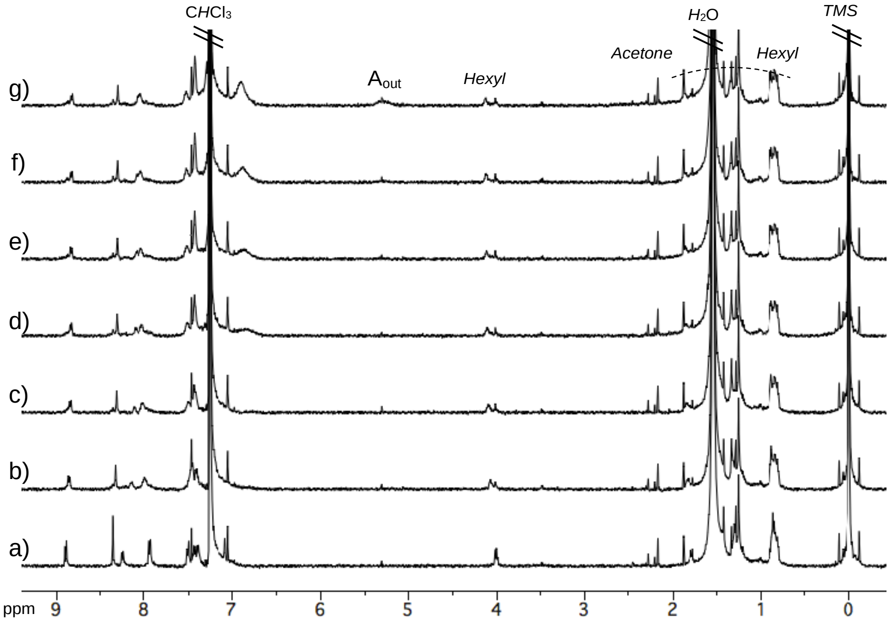

Fig. S10. ^{1}H NMR spectra of $[Ag_2\mathbf{L1}X_2](SbF_6)_2$ (0.07 mM) in the presence of a) 0.0, b) 1.0, c) 2.0, d) 3.0, e) 4.0, f) 5.0, and g) 7.0 eq of **Trip** (500 MHz, $CDCl_3$, 300 K).

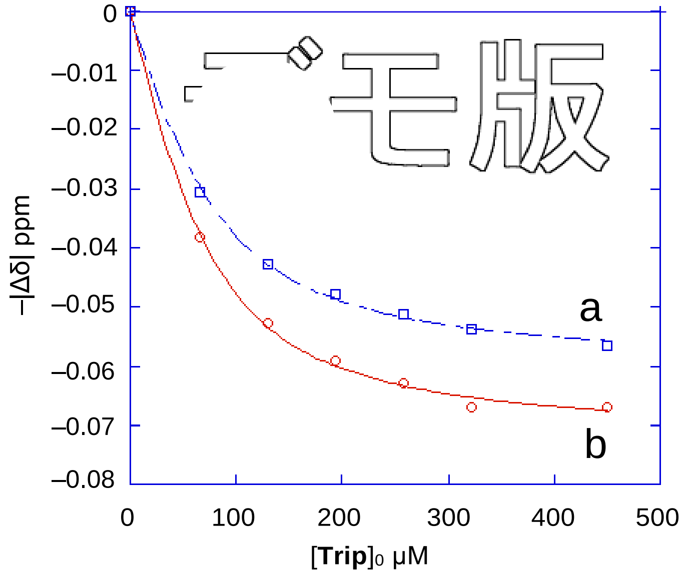

$$K_a(\mathbf{Trip}) = \frac{[\mathbf{Trip}\subset[Ag_2\mathbf{L1}]^{2+}]}{[\mathbf{Trip}]\,[[Ag_2\mathbf{L1}X_2]^{2+}]}$$

$$= (3.1 \pm 0.2) \times 10^4\ M^{-1}$$

in $CDCl_3$ at 300 K

Fig. S11. Stability constant analysis by the least square fitting to the shift of NMR signals ($H_{a,b}$) in the titration experiment described in Figs. S9–S10 (solid circles: observed, lines: calculated). $[\mathbf{Trip}]_0$ indicates the initial concentration of **Trip**.

^{1}H NMR titration experiment at 220 K

To a solution of $[Ag_2\mathbf{L1}X_2](SbF_6)_2$ in $CDCl_3$ (0.11 mM, 450 μL, 0.05 μmol, 1.0 eq) was added a solution of triptycene (**Trip**) in $CDCl_3$ (40 mM).

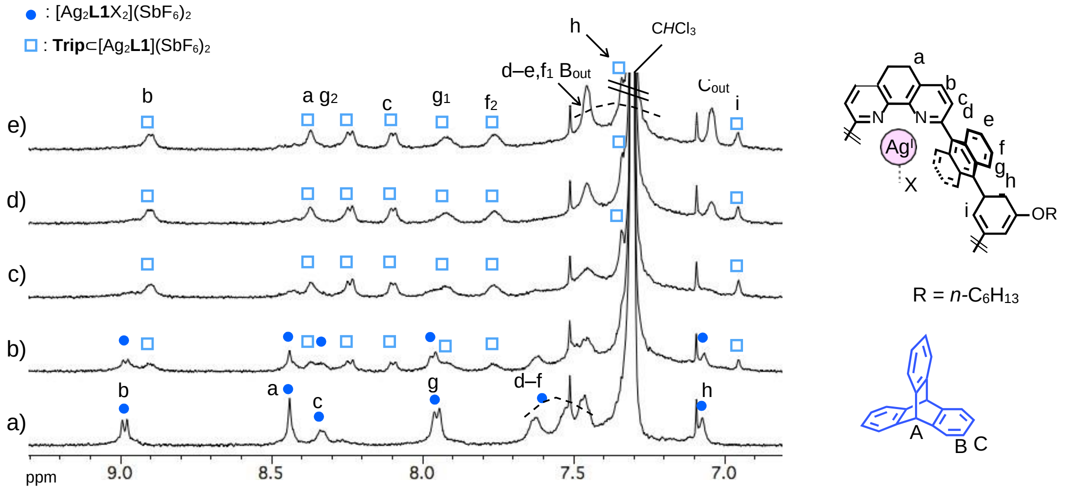

Fig. S12. Partial ^{1}H NMR spectra of $[Ag_2\mathbf{L1}X_2](SbF_6)_2$ (0.11 mM) in the presence of a) 0.0, b) 0.5, c) 1.0, d) 1.5, and e) 2.0 eq of **Trip** (500 MHz, $CDCl_3$, 220 K).

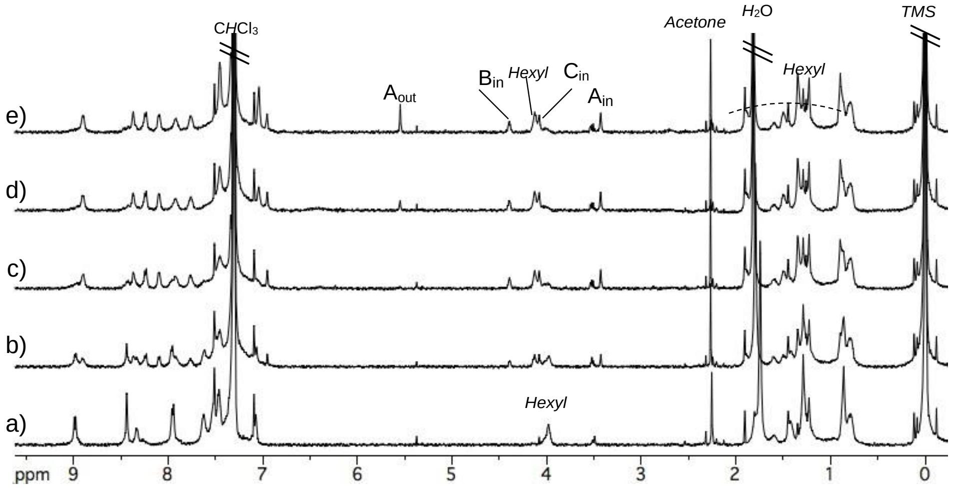

Fig. S13. ^{1}H NMR spectra of $[Ag_2\mathbf{L1}X_2](SbF_6)_2$ (0.11 mM) in the presence of a) 0.0, b) 0.5, c) 1.0, d) 1.5, and e) 2.0 eq of **Trip** (500 MHz, $CDCl_3$, 220 K).

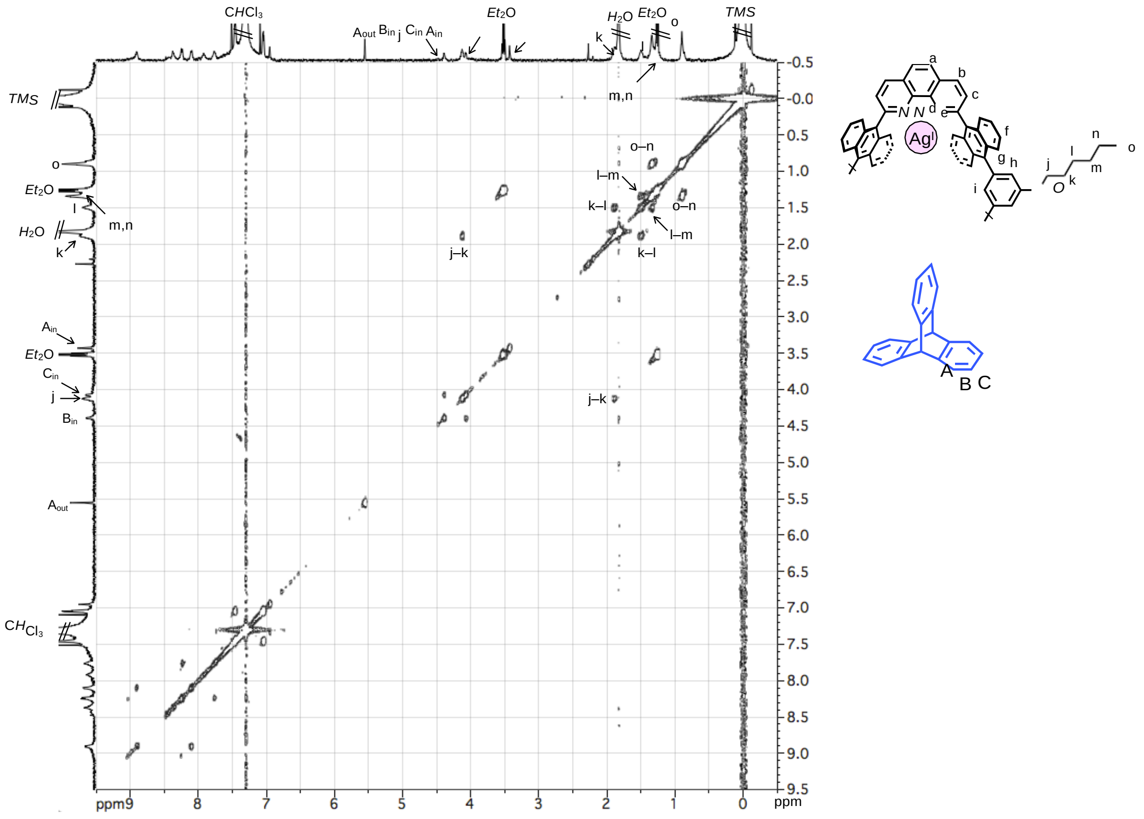

Fig. S14. Partial ^{1}H–^{1}H COSY spectrum of a mixture of $[Ag_2\mathbf{L1}X_2](SbF_6)_2$ (0.07 mM) and **Trip** (2.0 eq) (500 MHz, $CDCl_3$, 220 K).

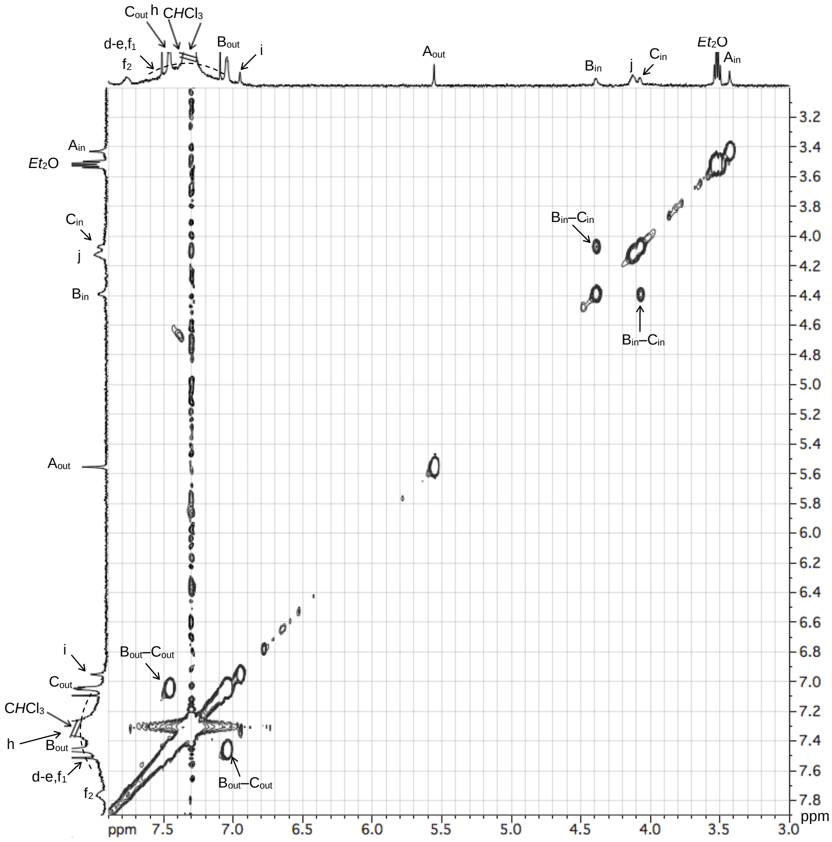

Fig. S15. Partial ^{1}H–^{1}H COSY spectrum of a mixture of $[Ag_2$**L1**$X_2](SbF_6)_2$ (0.07 mM) and **Trip** (2.0 eq) (500 MHz, $CDCl_3$, 220 K).

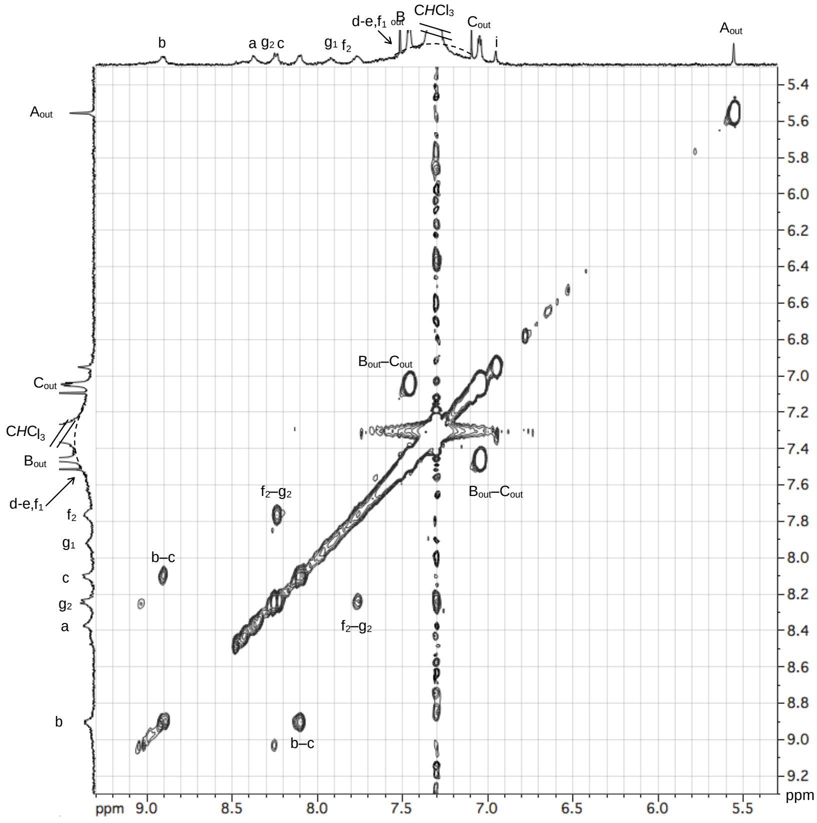

Fig. S16. ^{1}H–^{1}H COSY spectrum of a mixture of $[Ag_2$**L1**$X_2](SbF_6)_2$ (0.07 mM) and **Trip** (2.0 eq) (500 MHz, $CDCl_3$, 220 K).

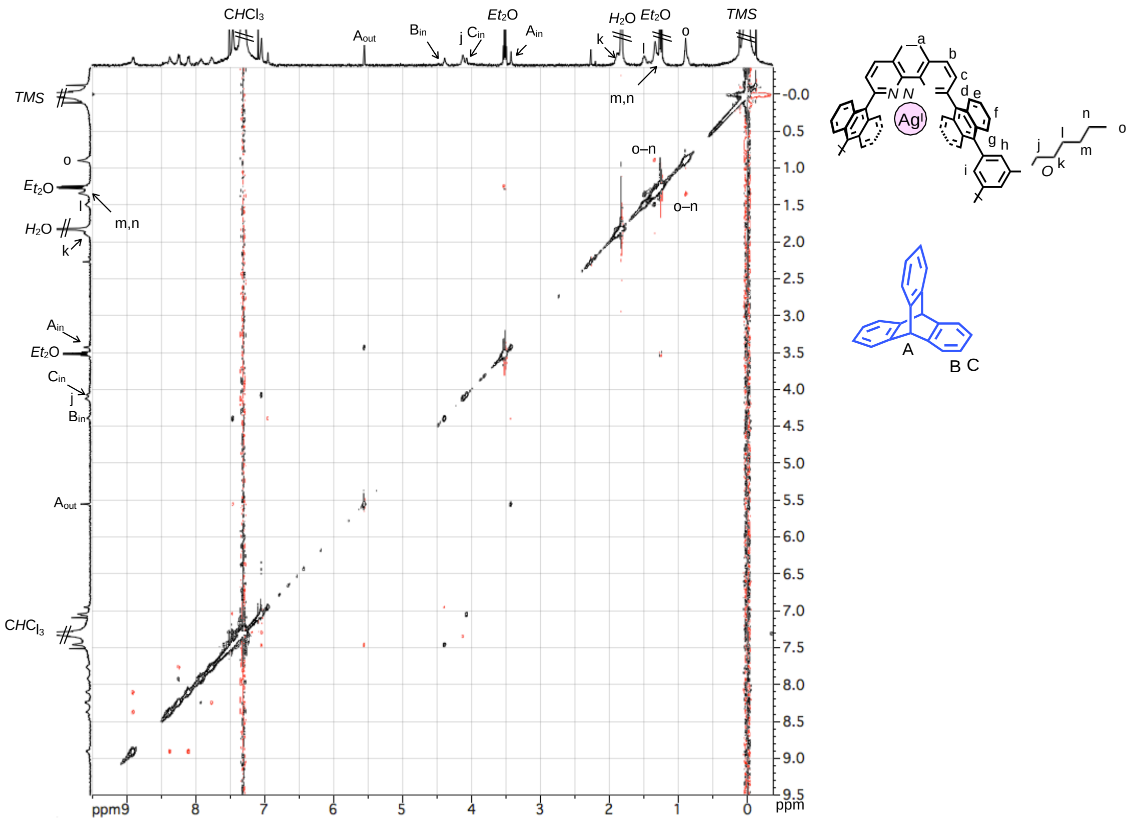

Fig. S17. Partial ^{1}H–^{1}H ROESY spectrum of a mixture of $[Ag_2\mathbf{L1}X_2](SbF_6)_2$ (0.07 mM) and **Trip** (2.0 eq) (500 MHz, $CDCl_3$, 220 K).

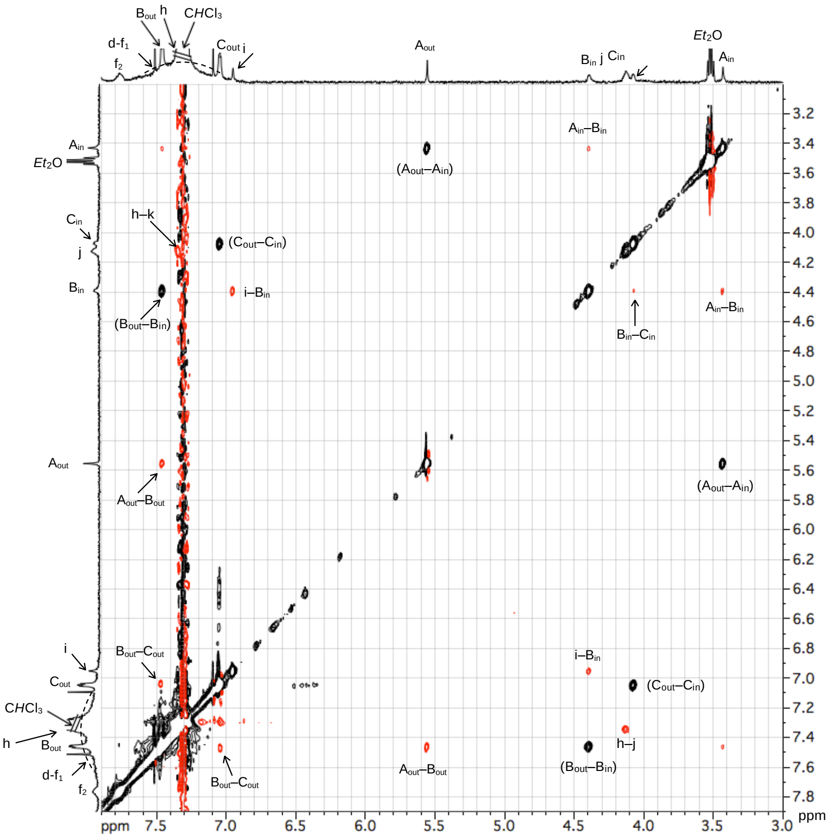

Fig. S18. Partial ^{1}H–^{1}H ROESY spectrum of a mixture of $[Ag_2\mathbf{L1}X_2](SbF_6)_2$ (0.07 mM) and **Trip** (2.0 eq) (500 MHz, $CDCl_3$, 220 K). Peaks assigned in parentheses originate from chemical exchange between free and included **Trip**.

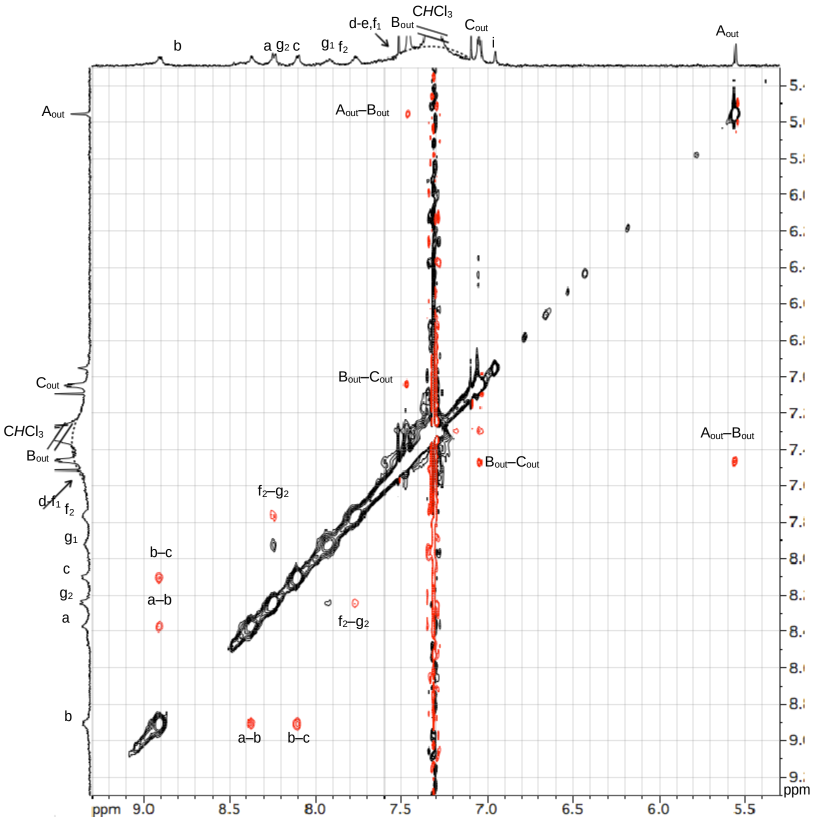

Fig. S19. Partial ^{1}H–^{1}H ROESY spectrum of a mixture of [Ag_2**L1**X_2](SbF_6)$_2$ (0.07 mM) and **Trip** (2.0 eq) (500 MHz, $CDCl_3$, 220 K).

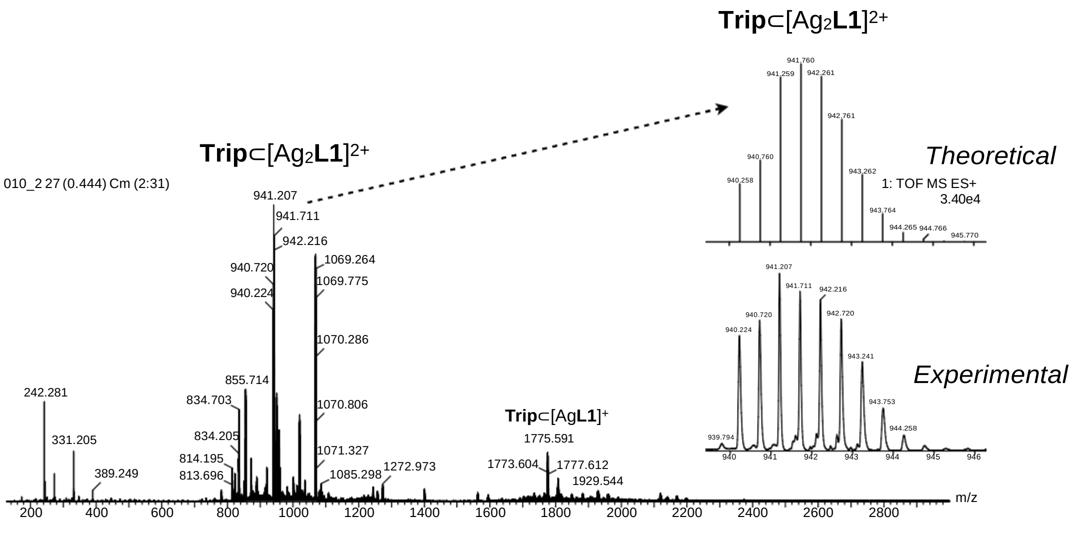

Fig. S20. ESI-TOF mass spectrum of a mixture of [Ag_2**L1**X_2](SbF_6)$_2$ and 7.0 eq of **Trip**.

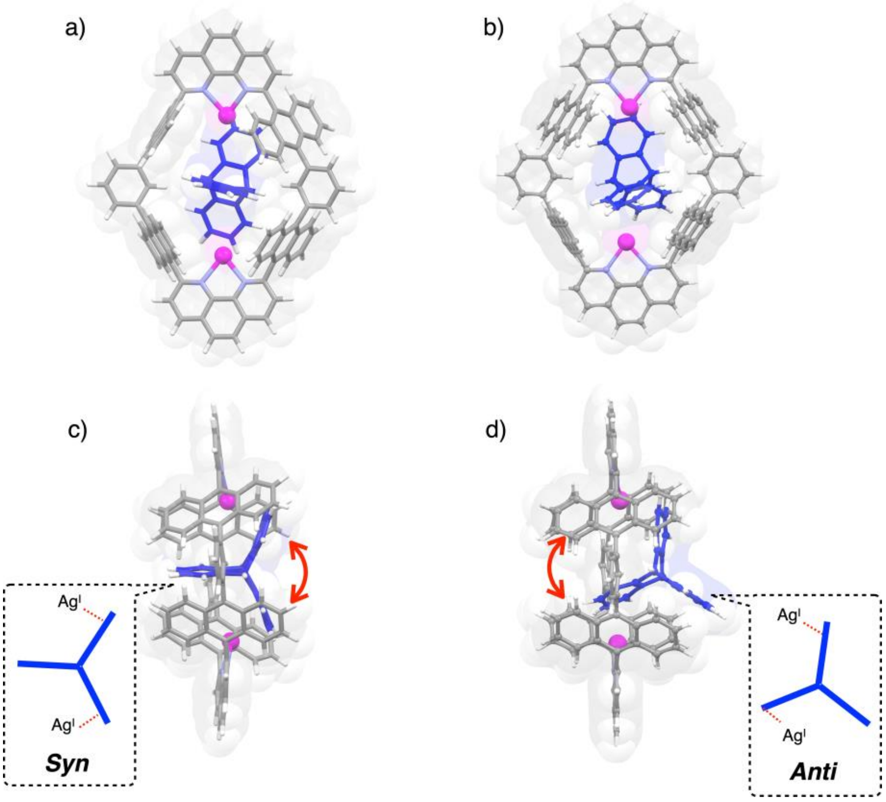

Fig. S21. Possible structures of **Trip**⊂ [Ag$_2$**L1**]$^{2+}$ based on molecular mechanics calculation; a) a front view and c) a side view of a *syn*-isomer; b) a front view and d) a side view of an *anti*-isomer. (Ag: magenta, C: grey, C of **Trip**: blue, H: white, N: blue). Side alkyloxy chains of **L1** are omitted for clarity. Red arrows in Fig. S21c–d represent a possible rotational movement of **Trip** within the nano-space of [Ag$_2$**L1**]$^{2+}$, which causes conservation of 3-fold rotational symmetry of **Trip** in the ^{1}H NMR time scale (Fig. S13).

Complexation of [Ag$_2$L1X$_2$](SbF$_6$)$_2$ and naphthalene

^{1}H NMR titration experiment

To a solution of [Ag$_2$**L1**X$_2$](SbF$_6$)$_2$ in CDCl$_3$ (0.11 mM, 475 μL, 0.052 μmol, 1.0 eq) was added a solution of naphthalene in CDCl$_3$ (20 mM).

Complexation of [Ag$_2$L1X$_2$](SbF$_6$)$_2$ and *p*-xylene

^{1}H NMR titration experiment

To a solution of [Ag$_2$**L1**X$_2$](SbF$_6$)$_2$ in CDCl$_3$ (0.11 mM, 475 μL, 0.052 μmol, 1.0 eq) was added a solution of *p*-xylene in CDCl$_3$ (9.7 mM).

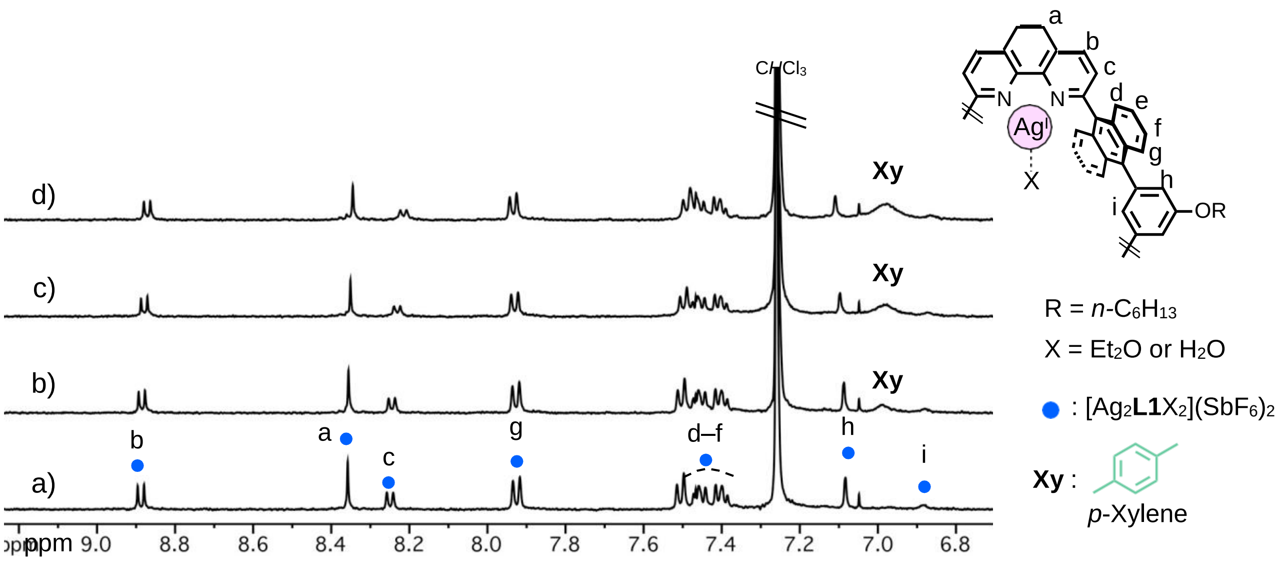

Fig. S22. Partial ^{1}H NMR spectra of [Ag$_2$**L1**X$_2$](SbF$_6$)$_2$ (0.11 mM) in the presence of a) 0.0, b) 1.0, c) 3.0, and d) 5.0 eq of *p*-xylene (500 MHz, CDCl$_3$, 300 K).

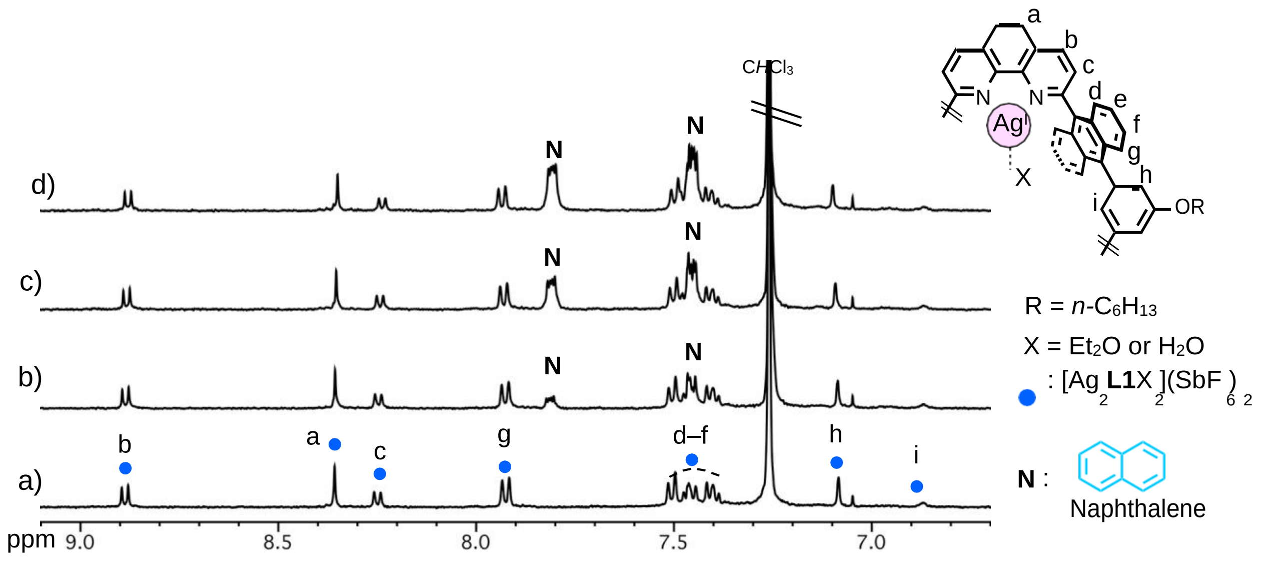

Fig. S23. Partial ^{1}H NMR spectra of [Ag$_2$**L1**X$_2$](SbF$_6$)$_2$ (0.11 mM) in the presence of a) 0.0, b) 1.0, c) 3.0, and d) 5.0 eq of naphthalene (500 MHz, CDCl$_3$, 300 K).

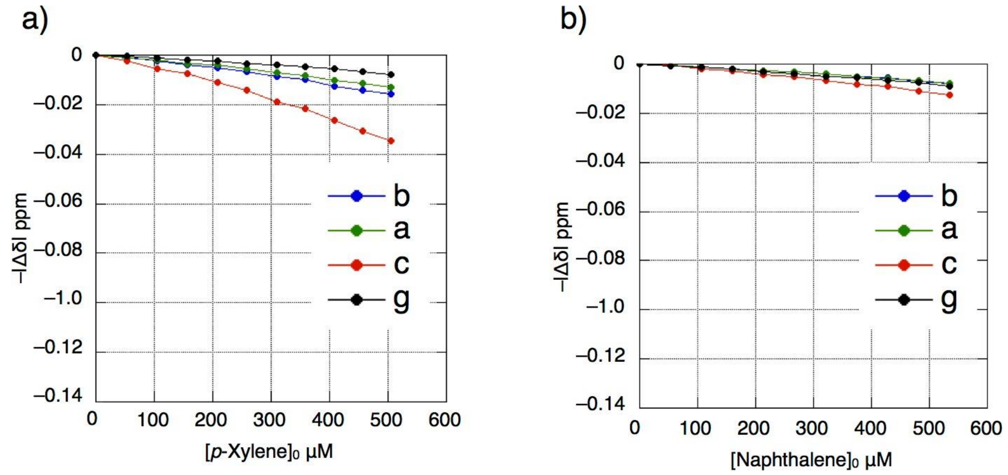

Fig. S24. Plots of the amounts of shift change of the ^{1}H NMR signals ($H_{a–c,g}$) against the concentrations of a) *p*-xylene and b) naphthalene. The spectra are shown in Figs. S22–S23. [*p*-Xylene]$_0$ and [Naphthalene]$_0$ indicate the initial concentrations of *p*-xylene and naphthalene, respectively.

Upon addition of *p*-xylene or naphthalene to a solution of [Ag$_2$**L1**X$_2$](SbF$_6$)$_2$ (0.11 mM) in CDCl$_3$, ^{1}H NMR signals of [Ag$_2$**L1**X$_2$](SbF$_6$)$_2$ at the aromatic region slightly shifted, but did not converge even in the presence of more than 5.0 eq of guests (Figs. S22–S23). Such almost stationary ^{1}H NMR spectra during titration experiments suggest negligible host-guest interactions or a different binding mode from the 1:1 host-guest structure of **Ant**⊂[Ag$_2$**L1**]$^{2+}$ or **Trip**⊂[Ag$_2$**L1**]$^{2+}$. It should be noted that in the case of titration experiment using **Ant** or **Trip** as guests, the shift of the signals almost converged under the same condition (Figs. S1–S2 and S9–S10).

4. References

1. K. Omoto. S. Tashiro, M. Kuritani and M. Shionoya, *J. Am. Chem. Soc.* 2014, **136**, 17946–17949.
2. G. M. Sheldrick, *SHELXL-97, Program for refinement of crystal structures*, University of Göttingen, Germany, 1997.
